# Analytical framework for understanding twisted 2D materials

**DOI:** 10.1093/nsr/nwag339

**Published:** 2026-06-13

**Authors:** Julia R Greer

**Affiliations:** Division of Engineering and Applied Sciences, California Institute of Technology, USA

The discovery of unconventional superconductivity in twisted bilayer graphene has ignited intense interest in twisted two-dimensional (2D) materials [1]. These systems exhibit remarkable quantum properties arising from Moiré superlattices, that is, periodic patterns formed when atomic layers are rotated relative to each other. A dearth of analytical models that can connect nanoscale deformation to the twist angle has limited our ability to predict and control these properties. In a recent study published in *National Science Review*, Li and colleagues [2] make significant strides to minimize this fundamental gap by developing a comprehensive theoretical framework that quantifies the salient mechanical attributes of twisted 2D lattices: strain, stress, rotation fields, and energetics.

The key insight of this work lies in recognizing that at small twist angles, Moiré patterns are dominated by special patterns connected by partial screw dislocations that enclose triangular regions with AB/BA stacking. The authors applied dislocation theory to derive analytical expressions for local rotation, strain, and stress fields within twisted 2D materials. Their model elegantly captures how neighboring layers undergo counterrotation in energetically favorable AB/BA stacking regions, while AA stacking regions shrink into localized nodes during reconstruction.

A particularly interesting result is that the authors developed a nonlinear relationship between the energy density and twist angle that captures the transition from rapid growth to near saturation across the angular range of 0–30°. At small twist angles (θ < θ_c_, where θ_c_ ≈ 2° for bilayer graphene), the energy is dominated by the triangular dislocation networks. At larger angles, the Moiré superlattice transforms into hexagonal domains with AA stacking separated by domain walls, resulting in a different energy landscape. This dual-regime model provides a unified description across the entire twist-angle range.

The theoretical predictions agree with the authors' large-scale atomistic simulations for twisted bilayer graphene (tB-G), twisted bilayer hexagonal boron nitride (tB-hBN), and twisted trilayer graphene (tT-G), with the predicted strain fields also matching four-dimensional scanning transmission electron microscopy measurements by Kazmierczak *et al.* [3]. These independent consistencies between the presented theory and computational results, as well as experimental observations, serve to validate the model for use in more complicated layered periodic media.

This work makes several important contributions. First, it provides the first complete analytical solutions for deformation fields in twisted 2D materials, moving beyond existing numerical approaches [4,5]. Second, the identification of the critical twist angle θ_c_ offers a quantitative criterion for distinguishing small-angle and large-angle regimes. Third, the framework is generalizable and applicable to single-element crystals, binary compounds, and multilayer systems.

From a practical perspective, this analytical model provides key insights into the rational design of twisted 2D quantum devices. Since electronic band structure and correlated phases are exquisitely sensitive to local strain and rotation [1,6], the ability to predict these fields solely from twist angle provides an essential guide for twist-angle engineering. The work also establishes connections between dislocation mechanics and 2D materials physics, opening avenues for applying classical defect theory to emerging quantum materials.
